# Single-Chain Variable Fragments: Targeting Snake Venom Phospholipase A_2_ and Serine Protease

**DOI:** 10.3390/toxins17020055

**Published:** 2025-01-24

**Authors:** Ying Jia, Ariane Garcia, Elizabeth Reyes

**Affiliations:** School of Integrative Biological and Chemical Sciences, University of Texas Rio Grande Valley, Brownsville, TX 78520, USA; ariane.garcia@utrgv.edu (A.G.); elizabeth.reyes@utrgv.edu (E.R.)

**Keywords:** snakebite, PLA_2_, serine protease, peptide, immunogenicity, single-chain variable fragment, immunoglobulin G

## Abstract

Snakebite is a critical global public health issue, causing substantial mortality and morbidity, particularly in tropical and subtropical regions. The development of innovative antivenoms targeting snake venom toxins is therefore of paramount importance. In this study, we adopted an epitope-directed approach to design three degenerate 15-mer peptides based on amino acid sequence alignments of snake venom phospholipase A_2_s (PLA_2_s) and snake venom serine proteases (SVSPs) from snake (*Crotalus atrox*). By leveraging their immunogenic and inhibitory profiles, these peptides were specifically designed to target the Asp49 and Lys49 variants of PLA_2_ and SVSP toxins. Groups of five mice were immunized with each peptide, and IgG mRNA was subsequently extracted from peripheral blood mononuclear cells (PBMCs) and spleen lymphocytes of the top three responders. The extracted mRNA was reverse-transcribed into complementary DNA (cDNA), and the variable regions of the IgG heavy and kappa chains were amplified using polymerase chain reaction (PCR). These amplified regions were then linked with a 66-nucleotide spacer to construct single-chain variable fragments (scFvs). Sequence analysis of 48 randomly selected plasmids from each PLA_2_ and SVSP scFv library revealed that over 80% contained scFv sequences with notable diversity observed in the complementarity-determining regions (CDRs), particularly CDR3. Enzyme-linked immunosorbent assay (ELISA) results demonstrated that the SP peptide elicited a broader immune response in mice compared to the Asp49 peptide, implying the strong immunogenicity of the SP peptide. These scFvs represent a promising foundation for the development of recombinant human monoclonal antibodies targeting snake PLA_2_ and SVSP toxins, providing a potential therapeutic strategy for the treatment of snakebites.

## 1. Introduction

Snakebite is a significant global public health concern, leading to high rates of mortality and morbidity, particularly in tropical and subtropical regions. The World Health Organization (WHO) has recognized the severity of the issue and classified snakebite envenomation as a category A neglected tropical disease (NTD) [1,2]. Current strategies for snakebite management primarily rely on antivenoms, which are polyclonal antibody-based therapeutics derived from animal serum; however, these polyclonal antivenoms face notable limitations, especially in addressing the wide variability in venom composition across different snake species, which complicates the development of effective treatments. Despite the complexity of snake venoms, the majority of medically significant venom toxins belong to a limited number of toxin families [3,4,5]. The predominant venom toxins in the three major recognized and medically important snake families, Elapidae, Viperidae, and Crotalidae, include phospholipase A_2_ (PLA_2_), snake venom metalloproteinase (SVMP), snake venom serine protease (SVSP), and three-finger toxin (3FTx) [6]. Among these, the broadly neutralizing antibodies targeting 3FTx in elapid snakes have been extensively developed [7,8,9]. To explore alternative therapeutic approaches, this research focuses on developing single-chain variable fragment (scFv) antibodies specifically targeting PLA_2_ and SVSP toxins.

The scFv is an engineered antibody fragment comprising the variable regions of the heavy (V_H_) and light (V_L_) chains of IgG antibody, linked by a short, flexible peptide typically composed of glycine and serine residues. This linker allows the V_H_ and V_L_ domains to fold into a functional antigen-binding site while increasing solubility through dispersed hydrophilic residues [10]. Both V_L_-linker-V_H_ and V_H_-linker-V_L_ configurations are capable of forming functional scFvs, though individual scFvs may exhibit enhanced performance in one configuration over the others [11]. Due to their high specificity and affinity, low immunogenicity, ease of engineering, scalability, and adaptability to diverse platforms, scFvs have gained wide application in medical, diagnostic, and research fields. For example, their unique structural and functional advantages over full-length monoclonal antibodies have positioned scFvs as critical tools in cancer diagnostics and therapeutics [12,13]. Remarkably, scFvs account for approximately 35% of all antibody fragments currently in clinical evaluation for various cancers, including breast, lung, and hematologic malignancies [14,15]. Clinically, the small size of scFvs confers advantages such as rapid clearance from blood, ideal for imaging applications, and superior solid tissue penetration, enhancing their utility in therapeutic and imaging contexts [16]. Additionally, scFvs are less immunogenic in vivo due to the absence of an Fc region [17,18]. Their versatility is further demonstrated by their ability to be conjugated with radionuclides or cytotoxic drugs, enabling precision-targeted therapies that minimize off-target effects [17]. However, scFvs also present challenges, including reduced long-term stability, lower affinities compared to full-length antibodies, and a propensity for aggregation due to their small size [18]. For instance, their rapid clearance from circulation, with a half-life of 0.5–2 h, limits their retention at target sites [13]. Despite these limitations, this study explores the potential of scFvs targeting PLA_2_ and SVSP from the venom of the western diamondback rattlesnake (*Crotalus atrox*).

The western diamondback rattlesnake (*C. atrox*), a prominent member of the Crotalidae family, is widely distributed across North America and is a leading cause of snakebite fatalities in northern Mexico and the second highest number in the United States [19]. More than 70% of adult *C. atrox* venom comprises PLA_2_, SVMP, and SVSP [20]. Accordingly, this study utilizes the primary amino acid sequences of PLA_2_ and SVSP from *C. atrox* [21] to design toxin-targeting peptides. PLA_2_s are a key venom toxin implicated in tissue damage and exhibit diverse pathophysiological effects, including hemorrhagic, myotoxic, hypotensive, presynaptic, cardiotoxic, hemolytic, and anticoagulant activities, mediated through both catalytic and non-catalytic mechanisms [22,23]. PLA_2_ constitutes more than 7% of *C. atrox* venom, with at least two distinct transcripts identified in its venom and venom glands [20,21,24]. Similarly, SVSPs, prevalent in viperid and crotalid venoms, disrupt hemostatic balance by enzymatically targeting coagulation, fibrinolytic, and kallikrein–kinin systems [25]. These proteolytic enzymes, which feature the classical Ser-His-Asp catalytic triad [26], possess hemostatic and hemorrhagic effects [27,28]. Immunoreactivity studies, such as those by Madrigal et al. [29], have identified promising immunogenic peptides derived from SVSP.

To develop broadly neutralizing antivenoms, this research employs the primary amino acid sequences of Asp49 and Lys49 PLA_2_ isoforms and SVSP from *C. atrox* as templates for scFv design, synthesis, and immunization. This approach aims to identify potent interactions between IgG variable regions and major venom toxins, ultimately facilitating the development of recombinant oligoclonal antibodies to neutralize snake venom toxins effectively.

## 2. Results and Discussion

### 2.1. Peptide Design and Synthesis

**Figure 1:**
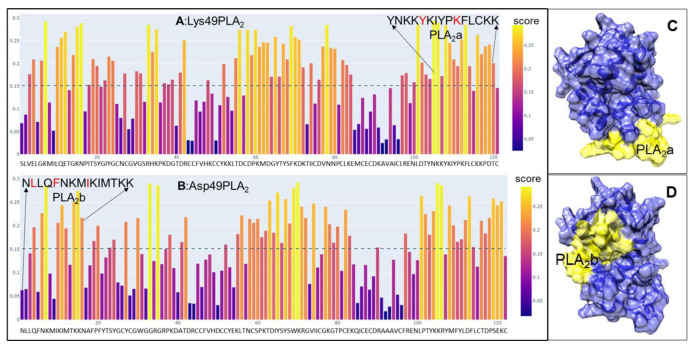
Epitope prediction for PLA_2_ proteins. (**A**) Predicted epitope (PLA_2_a) containing functional amino acids (in red) for Lys49 PLA_2_. (**B**) Predicted epitope (PLA_2_b) containing functional amino acids (in red) for Asp49 PLA_2_. (**C**) Three-dimensional structure of Lys49 PLA_2_ exhibiting that PLA_2_a is accessible. (**D**) Three-dimensional structure of Asp49 PLA_2_ displaying that PLA_2_b is accessible.

**Figure 2:**
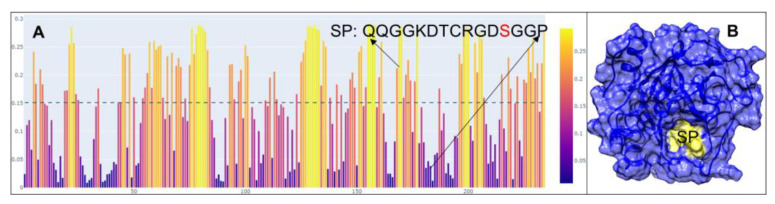
Predicted epitope for SVSP. (**A**) Predicted epitope (SP) containing the functional amino acid “Ser”. (**B**) Simulated 3D structure of SVSP showing the SP is exposed and accessible (**B**).

**Figure 3:**
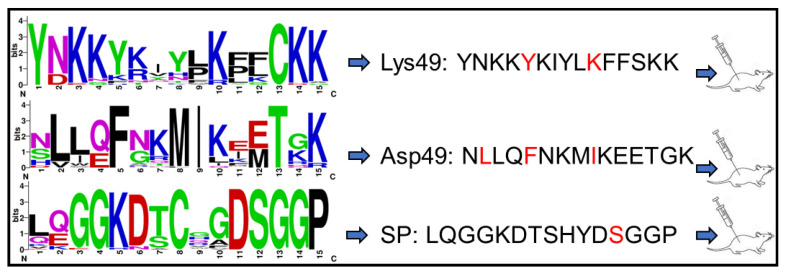
Generation of degenerate PLA_2_ and SVSP peptides. Multiple sequence alignments were generated using NCBI Protein BLAST and further analyzed with WebLogo to identify the conserved residues. The final peptides (Lys49, Asp49, and SP) were designed based on the conserved regions, slightly modified, synthesized, and then used for immunizing mice.

We utilized the amino acid sequences of two mature PLA_2_ isoforms, Lys49 and Asp49, cloned from *C. atrox* venom glands. These sequences are identical to NCBI entries Q8UVZ7 and APD70896, respectively. Using the online epitope prediction tool BepiPred-3.0 [30], we identified four major discontinuous antigenic epitopes for each PLA_2_ protein (Figure 1A,B). Among these, only the cationic C-terminal region of the Lys49 PLA_2_ protein, comprising 13 amino acid residues (KKYRYYLKPLCKK), is associated with its myotoxic activity [31]. Specifically, the residue Tyr117 and the positively charged Lys122, particularly Lys122, enhance the affinity for fatty acid head groups, thereby eliciting myotoxic effects [32,33]. In contrast, the Asp49 PLA_2_ isoform exhibits an N-terminal peptide (NLLQFNKMIKIMTKK) with both immunogenic and neutralization properties [34,35]. Residues such as Leu2, Phe5, and Ile9 within this region are critical for substrate interactions [36]. These two epitopes are surface-exposed and accessible in the AlphaFold-simulated 3D structures (Figure 1C,D). We consequently selected these segments, referred to as PLA_2_a for Lys49 and PLA_2_b for Asp49, for further peptide design. To develop degenerate peptides for potential broad-spectrum scFvs targeting diverse snake venom PLA_2_s, we conducted multiple sequence alignments using PLA_2_ sequences from 75 snake species across the Elapidae, Crotalidae, and Viperidae families. The regions corresponding to PLA_2_a and PLA_2_b across these sequences were further analyzed to identify the conserved amino acids, after which the final degenerate peptides were designed with slight modifications, such as substituting “Cys” with “Ser” and incorporating “Tyr” residues in peptides lacking aromatic amino acids, to prevent disulfide bond formation and facilitate conjugation. A similar approach was applied to identify a 15-amino acid peptide, termed SP, from *C. atrox* serine proteases (SVSPs) (Figure 2). The SVSP sequence matched 100% with NCBI entry AUS82489. We conducted a BLAST search using the SVSP sequence and performed multiple alignments with SVSP sequences from 30 snake species to identify conserved residues (Figure 3). This 15-mer peptide was then slightly modified using the same strategy for PLA_2_ and finalized for immunization (Figure 3).

### 2.2. Animal Immunization

**Figure 4:**
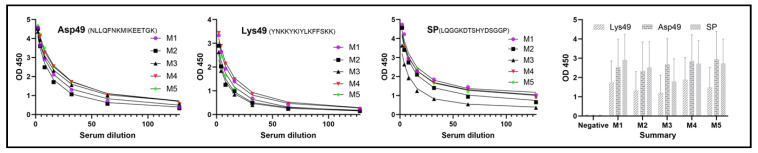
Animal immunization. A group of five BALB/c mice was immunized with each peptide at a dose of 0.25 mg, followed by three booster injections of 0.25 mg each. Immunogenicity was assessed via ELISA using serum extracted from each mouse. The top three mice (e.g., mice 1, 4, and 5) with the strongest immune responses were selected for the isolation of peripheral blood mononuclear cells (PBMCs) and spleen lymphocytes. Serum was diluted at the following concentrations: 1:2000, 1:4000, 1:8000, 1:16,000, 1:32,000, 1:64,000, and 1:128,000. Negative: serum collected prior to peptide immunization.

Two groups of five mice were immunized with either a mixture of Lys49 and Asp49 peptides or with SP peptide alone, and serum titers were individually evaluated using an enzyme-linked immunosorbent assay (ELISA) against the corresponding peptide. Overall, the peptide Asp49 (NLLQFNKMIKEETGK) derived from the Asp49 PLA_2_ enzyme and SP (LQGGKDTSHYDSGGP) from serine protease elicited similar immune responses, both of which were stronger than the response to Lys49 (YNKKYKIYLKFFSKK) from the Lys49 PLA_2_ protein (Figure 4). Furthermore, all mice displayed comparable immunoreactivity to each peptide, while mice M1, M4, and M5 in each group showed slightly stronger immune responses (Figure 4). Consequently, these mice (M1, M4, and M5) were selected for PBMC and lymphocyte isolation.

### 2.3. Characterization of scFv

**Figure 5:**
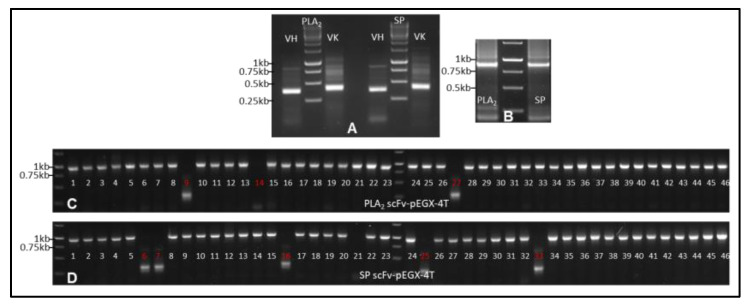
Generation of scFvs. (**A**) The variable regions of the IgG heavy and kappa chains were individually amplified from the cDNA of PBMC and lymphocytes immunized with Asp49, Lys49, or SP peptides. (**B**) The variable regions of the heavy and kappa chains were linked via a 66-nucleotide linker to generate the scFvs for PLA_2_ and SP. (**C**,**D**) Randomly selected 48 plasmid DNAs were analyzed to check for inserts and sequencing.

The scFvs generated through phage display technology have been extensively utilized to develop effective reagents for both therapeutic and diagnostic applications [37,38]. However, phage display experiments are not only time-consuming and labor-intensive but also present significant challenges, including limited binding affinity and the generation of false positives [39,40]. These limitations often necessitate specialized technical expertise [41,42]. In contrast, our streamlined procedure for generating and screening scFv clones provides a renewable resource characterized by versatility, scalability, and consistency. Furthermore, harnessing the advantages of scFvs allows us to deepen our understanding of toxin–antibody interactions and explore novel pathways for the development of therapeutic antivenoms. Therefore, using degenerate primers (Table 1), we successfully amplified the variable regions of both the heavy and kappa light chains from IgG cDNA derived from PLA_2_- and SP-peptide-immunized samples. In BALB/c mice, over 95% of light chains are kappa, with less than 5% being lambda [43]. Therefore, this study focused exclusively on the kappa light chain. The amplified variable regions were approximately 350 bp for the heavy chain and 400 bp for the kappa chain (Figure 5A). These regions were subsequently ligated via a 66 bp double-stranded DNA fragment encoding a peptide linker (VSSGGGGSGGGGSGGGGSDIEL) to produce scFvs in the configuration heavy-linker-kappa for both PLA_2_ and SP, yielding scFvs of approximately 810 bp (Figure 5B). The scFv constructs for PLA_2_ and SP were ligated into a GST-tagged expression vector (pEGX-4T) and introduced into *E. coli* BL21 cells, generating scFv libraries with titers of 1.1 × 10^7^ cfu/mL for PLA_2_ and 3.5 × 10^7^ cfu/mL for SP.

**Figure 6:**
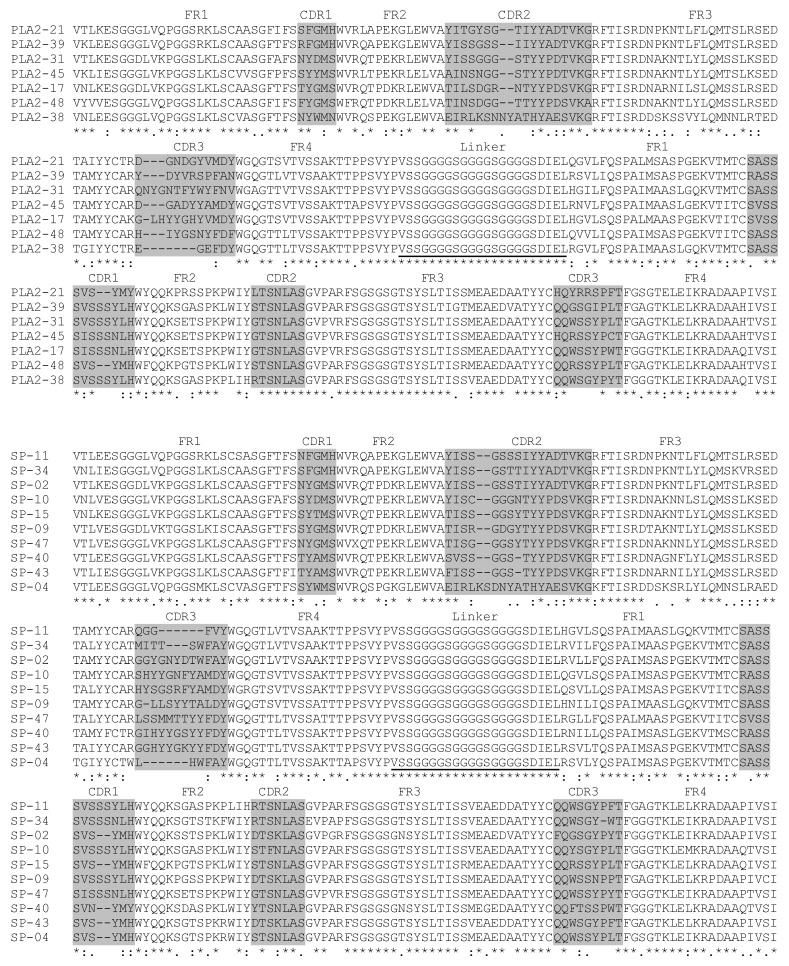
Amino acid sequence alignment of scFvs. The upper panel (PLA_2_) shows PLA_2_ scFv sequence alignment, while the lower panel (SP) reveals the SP scFv sequence alignment. The complementarity-determining regions (CDR) and framework regions (FR) were labeled in grey and connected through a linker underlined to make the (V_H_-linker-V_L_) configuration.

To evaluate the quality of the libraries, 48 clones were randomly selected from each library, and their inserts were amplified. The results showed that 84% of the PLA_2_ scFv library clones and 90% of the SP scFv library clones contained inserts (Figure 5C,D). These clones were further sequenced, and their affinities to peptides were assessed using the ELISA method. In the sequenced 48 clones of the PLA_2_ scFv library, 37 sequences encoded mouse heavy and light chains, including 7 perfect sequences (Figure 6) and 30 sequences requiring editing based on database comparisons and trace file analysis, while the remaining sequences were unreadable. Each of the 37 PLA_2_ scFv sequences was unique, with differences of more than three amino acids. In the sequenced 48 clones of the SP scFv library, 38 sequences encoded heavy and light chains, comprising 10 perfect sequences (Figure 6) and 28 requiring editing, while the rest were unreadable. Among the SP scFv sequences, four were identified, with two being identical, while only two redundant clones were found between the sequenced 48 clones in the PLA_2_ and SP scFv libraries.

Each of the two variable domains of scFv contains three hypervariable or complementarity-determining regions (CDRs) interlinked by framework regions (FRs) (Figure 6). The CDRs of the heavy chain (V_H_) and light chain (V_L_) were identified following the method described by Kabat et al. [44]. The CDRs are primarily responsible for antigen binding, with their structures complementing specific epitopes. In contrast, the FRs serve as scaffolds with minimal variability compared to the CDRs. Among the CDRs, CDR3 in both the heavy and light (kappa) chains exhibit greater diversity than CDR1 and CDR2 (Figure 6). Wilson et al. [45] also demonstrated that the contribution of each CDR to antigen binding varies, with the heavy chain CDR3 playing a pivotal role by contributing 29% to lineage specificity, whereas CDR2L contributes only 4%.

**Figure 7:**
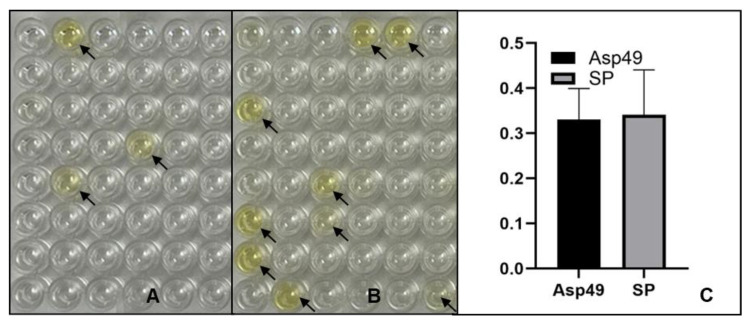
ELISA for detection of peptide-scFv interactions. Half of a 96-well plate was coated with Asp49 peptide (**A**), while the other half was coated with SP peptide (**B**). (**C**) A summary of binding affinity. Each of the 48 sequenced PLA_2_ and SP scFvs clones was incubated with Asp49 and SP peptide, respectively. Anti-GST primary antibody and secondary antibody conjugated with HRP were used for detection in the ELISA. Arrows indicate positive interactions.

To evaluate whether the obtained scFv clones recognize PLA_2_ and SP peptides, each of the 48 sequenced clones targeting PLA_2_ and SP was tested for interactions with their respective peptides. ELISA results (Figure 7) revealed that only three PLA_2_ scFv clones interacted with Asp49 peptide, while at least nine SP scFv clones bound to SP peptide. These findings suggest that the SP peptide elicited a broader immune response in mice compared to the Asp49 peptide. However, there was no significant difference in binding affinity between the scFv clones for the Asp49 and SP peptides (Figure 7C). Additionally, we detected interactions of the GST tag only with Asp49, Lys49, and SP peptides, and no interactions were observed. Further screening of the scFv libraries using high-stringent washing methods is planned to identify clones with the highest binding affinities. These selected clones will subsequently be analyzed for their toxin-inhibitory activity in follow-up experiments.

## 3. Conclusions

Snakebite envenomation remains a significant public health issue, particularly in tropical regions. This research project explores an alternative approach to snakebite treatment by developing scFvs targeting the major snake venom toxins. We successfully produced scFvs against snake venom PLA_2_ and SVSP, two major venom components. Unlike polyclonal antibodies or hybridoma-derived monoclonal antibodies, scFvs offer the advantage of genetic manipulability, enabling improved specificity and affinity while also reducing production costs. In subsequent experiments, we plan to screen scFv libraries against individual peptide targets, identify high-affinity interactions, and evaluate the toxin-neutralizing capabilities of these scFvs against native PLA_2_ and SVSP isolated from the crude venom of various snake species. Ultimately, our goal is to generate recombinant human monoclonal antibodies to develop a broad-spectrum oligoclonal antibody therapy effective against a wide range of snake venoms.

## 4. Materials and Methods

### 4.1. Peptide Design

The venom of adult *C. atrox* comprises more than 7% PLA_2_ [20] with at least two distinct transcripts encoding PLA_2_ proteins, Asp49 and Lys49 isoforms, identified in the venom glands and crude venom [21,24]. The epitopes for these PLA_2_ proteins were predicted using the BepiPred-3.0 tool [28]. Multiple amino acid sequence alignments were generated using NCBI Protein BLAST and multiple alignment tools. The alignment results were then processed through WebLogo [46] to assess the amino acid sequence similarity and conservation within the predicted epitope regions, which informed the selection of degenerate peptide sequences for synthesis. To design and synthesize SVSP peptide, we followed the same procedure as that used for PLA_2_ peptides. Additionally, the three-dimensional structures of PLA_2_s and SVSP were modeled using AlphaFold [47] by accessing the Texas Advanced Computing Center (TACC). All peptides were synthesized and verified using mass spectrometry (MS) and high-performance liquid chromatography (HPLC) by Biomatik Corporation (Ontario, Canada) and were used as immunogens for mouse immunization experiments.

### 4.2. Animal Immunization

In collaboration with ProteoGenix (Schiltigheim, France), the animal experiments were conducted following a protocol approved by the University of Texas Rio Grande Valley (UTRGV) Institutional Animal Care and Use Committee (AUP-23-15). BALB/c mice, weighing 18–20 g, were acclimatized for one week before the initiation of the experiments. The mice were housed in standard cages under controlled conditions: ambient temperature maintained at approximately 22 °C, relative humidity between 40% and 50%, and a 12-hour light/dark cycle. Food and reverse osmosis water were provided *ad libitum* via an automated water delivery system. For immunization, five mice were used per peptide, with each receiving an initial dose of 0.25 mg of peptide, followed by three booster injections of the same amount. The efficacy of immunization was assessed by evaluating the ability of serum derived from immunized mice to recognize the respective peptides. From the group, three mice showing the strongest immune responses were selected for the isolation of peripheral blood mononuclear cells (PBMCs) and spleen lymphocytes. PBMCs and spleen lymphocytes from mice immunized with Asp49 or Lys49 peptides were pooled to extract IgG mRNA.

### 4.3. Enzyme-Linked Immunosorbent Assay

The ELISA was performed following the protocol described by Slutzki et al. [48], with modifications as detailed below: (1) A 96-well plate (Thermo Fisher Scientific, Waltham, MA, USA, 439454) was coated with 100 µL of peptide (10 µg/mL) prepared in 1× Boca coating buffer (Thermo Fisher Scientific, Waltham, MA, USA, NC1894485) and incubated overnight at 4 °C. (2) Single-chain variable fragment (scFv) clones were incubated in 1.5 mL tubes containing 1 mL of LB broth supplemented with ampicillin (100 µg/mL) and IPTG (1 mM) at 37 °C for two days with shaking (220 rpm). (3) The peptide-coated 96-well plate was washed three times with 200 µL of 1× PBS buffer, blocked with 150 µL of SuperBlock buffer (Thermo Fisher Scientific, Waltham, MA, USA, 37515) per well for 1 h with shaking, and then rinsed once with 200 µL of 1× PBS buffer. (4) Bacterial cultures from Step (2) were centrifuged, the supernatant discarded, and the cell pellet resuspended in 100 µL of 1× PBS buffer. After this, 10 µL of lysozyme (20 µg/µL) was added, followed by incubation at 37 °C with gentle shaking (100 rpm) for 1 h. Cell debris was then pelleted by centrifugation at top speed for 3 min. (5) A total of 100 µL of the bacterial lysate from Step (4) was added to the prepared 96-well plate from Step (3) and incubated overnight at 4 °C. For immunogenicity tests, sera from the three highest-responder mice were serially diluted (1:2000 to 1:128,000) and added to the 96-well plates. (6) The plate was washed three times with 200 µL of 1× PBS buffer. (7) The wells were incubated with 100 µL of anti-GST primary antibody (Sigma-Aldrich, St. Louis, MO, USA, G7781) diluted 1:10,000 in 5% BSA/TBST for 1.5 h at room temperature with gentle shaking, followed by three washes with 1× TBST buffer. (8) Subsequently, 100 µL of goat anti-rabbit secondary antibody conjugated with HRP (Thermo Fisher Scientific, Waltham, MA, USA, 32460), prepared in a 1:10,000 dilution in 5% BSA/TBST, was added. The plate was incubated with shaking for 1.5 h at room temperature and then washed three times with 1× TBST buffer. (9) A total of 100 µL of TMB-ELISA substrate solution (Thermo Fisher Scientific, Waltham, MA, USA, 34029) was added to each well and incubated in the dark until the solution turned blue (approximately 1 h). (10) The reaction was stopped by adding 100 µL of sulfuric acid buffer (Thermo Fisher Scientific, Waltham, MA, USA, 035663-k2). The absorbance was measured at 450 nm using a plate reader (Accuris Instruments, Edison, NJ, USA. R9610). The ELISA results were analyzed using GraphPad Prism software (version 10.2.3).

### 4.4. IgG Complementary DNA Generation

PBMCs and spleen lymphocytes were promptly isolated from the three most effectively immunized mice, which were euthanized following the UTRGV-approved protocol (AUP-23-15). Total RNA was extracted from the PBMCs and lymphocytes using TRIzo reagent (Thermo Fisher Scientific, Waltham, MA, USA, 15596026). The extracted RNA was subsequently reverse-transcribed into complementary DNA (cDNA) according to the manufacturer’s instructions for the Maxima First Strand cDNA Synthesis Kit (Thermo Fisher Scientific, Waltham, MA, USA, K1641).

### 4.5. scFv Generation

To generate single-chain variable fragments (scFvs), we began by extracting DNA sequences encoding mouse immunoglobulin G (IgG) heavy and kappa chains from the NCBI database. Multiple sequence alignments were performed separately for the heavy and kappa chain sequences. Based on these alignments, degenerate primers (Table 1) targeting the constant regions of IgG were designed to amplify the variable regions of both the heavy and kappa chains by polymerase chain reaction (PCR). PCR was performed using Phusion High-Fidelity DNA Polymerase (Thermo Fisher Scientific, Waltham, MA, USA, F530) with the following setup: after initial denaturation of DNA at 98 °C for 30 s, 35 cycles of amplification were performed, including denaturation at 98 °C for 10 s, annealing at 55 °C or 58 °C (55 °C for amplifying V_H_ and V_L_, while 58 °C for amplifying scFv) for 30 s, and extension at 72 °C for 1 min, followed by further extension for 10 min at 72 °C. PCR products, visualized in 1.5% agarose gel by staining with SYBR Safe DNA gel stain (Thermo Fisher Scientific, Waltham, MA, USA, S33102), were excised and purified using the GENECLEAN kit (Thermo Fisher Scientific, Waltham, MA, USA, 111001200).

To link the variable regions, a 66-nucleotide double-stranded linker was synthesized using sequences described by Wang et al. [49] and Lang et al. [50]. The variable heavy (V_H_) and variable light (V_L_) chains were joined (V_H_-linker-V_L_) using the In-Fusion Snap Assembly Master Mix (Takara, San Jose, CA, USA, 638947) at the molecular ratio of V_H_ to V_L_ to linker DNA of approximately 2:2:1. The assembled scFv constructs were directly used to amplify the scFvs by PCR and purified from agarose gels, digested with EcoR I and Xho I, and subsequently ligated into the EcoR I and Xho I sites of the pGEX-4T vector (Addgene, Watertown, MA, USA, 29567) using T4 DNA Ligase (BioLabs, Ipswich, MA, USA, 0202). Finally, the ligation products were transformed into *E. coli* BL21 (DE3) competent cells by electroporation for the generation of scFv libraries.

## Figures and Tables

**Table 1:** Primers for amplification of heavy, kappa, and scFv.

Primer	Target	Sequences
VhF	V_H_	GTKAMKCTRRWRGAGTCTGG
VhR	V_H_	TCCACCTGAGGAGACTGGATAGACHGATGGGGSTGT
VkF	V_L_	TCGGACATCGAGCTCCRMRDTVTKCTSWHHCARWCW
VkR	V_L_	GATRGATACARTKKGTGCAGCATC
Linker		gtctcctcaggtggaggcggttcaggcggaggtggctctggcggtggcggatcggacatcgagctc
scFv-EcoRI	scFv	GGT*GAATTC*GTKAMKCTRRWRGAGTCTGG
scFv-XhoI	scFv	GGT*CTCGAG*TCATTAGATRGATACART

Underlined sequences are for in-fusion ligation with linker, and restriction sites are italicized.

## Data Availability

The original contributions presented in this study are included in the article. Further inquiries can be directed to the corresponding author.
